# Uncovering heart failure with preserved ejection fraction in patients with type 2 diabetes in primary care: time for a change

**DOI:** 10.1007/s12471-016-0809-7

**Published:** 2016-02-23

**Authors:** L. J. M. Boonman-de Winter, M. J. Cramer, A. W. Hoes, F. H. Rutten

**Affiliations:** Department of Scientific and Contract Research, Center for Diagnostic Support in Primary Care (SHL-Groep), Bredaseweg 165, 4872 LA Etten-Leur, The Netherlands; Department of Cardiology, Heart-Lung Center, University Medical Center Utrecht, Utrecht, The Netherlands; Julius Center for Health Sciences and Primary Care, University Medical Center Utrecht, Utrecht, The Netherlands

**Keywords:** Heart failure, Type 2 diabetes, Primary care, Screening, Preserved ejection fraction

## Abstract

Undetected heart failure appears to be an important health problem in patients with type 2 diabetes and aged ≥ 60 years. The prevalence of previously unknown heart failure in these patients is high, steeply rises with age, and is overall higher in women than in men. The majority of the patients with newly detected heart failure have a preserved ejection fraction. A diagnostic algorithm to detect or exclude heart failure in these patients with variables from the medical files combined with items from history taking and physical examination provides a good to excellent accuracy. Annual screening appears to be cost-effective. Both unrecognised heart failure with reduced and with preserved ejection fraction were associated with a clinically relevant lower health status in patients with type 2 diabetes. Also the prognosis of these patients was worse than of those without heart failure. Existing disease-management programs for type 2 diabetes pay insufficient attention to early detection of cardiovascular diseases, including heart failure. We conclude that more attention is needed for detection of heart failure in older patients with type 2 diabetes.

## Introduction

At present, disease management of patients with type 2 diabetes (T2DM) is mainly focussed on glucose regulation, blood pressure and lipid control, prevention of diabetic ulcers, and the early detection of specific micro-vascular problems, typically albuminuria and retinopathy. Despite increased emphasis on lipid and blood pressure control in the last decade, premature cardiovascular diseases remain the main cause of mortality in patients with T2DM [1]. It is therefore surprising that current disease management programs do not yet prioritise early detection of cardiovascular diseases.

In view of this, we hypothesised that especially older patients with T2DM may have unsuspected and unrecognised heart failure. Especially heart failure with preserved ejection fraction (HFpEF) is expected to be highly prevalent, because in the early stages of this subtype of heart failure, symptoms often only occur after exercise, and signs of fluid retention can be inconspicuous. The risk of remaining undetected and being wrongly labelled as e.g. chronic obstructive pulmonary disease (COPD) is high for HFpEF, the more because echocardiography is not readily available in primary care, while echocardiography is essential for establishing the diagnosis of HFpEF.

## Heart failure in type 2 diabetes in the Netherlands

Our group showed that the prevalence of previously unknown heart failure in patients with T2DM aged ≥ 60 is high (27.7 %), steeply rises with age, and is overall higher in women than in men (31.0 vs. 24.8 %, respectively). The majority (83 %; i.e. 22.9 % of all T2DM patients aged ≥ 60 years) had HFpEF, while 17 % (i.e. 4.8 % of all T2DM patients ≥ 60 years) had previously unrecognised heart failure with reduced ejection fraction (HFrEF) [2]. To unmask early disease manifestations we developed a diagnostic algorithm to detect or exclude heart failure in T2DM patients aged ≥ 60 years [3]. Variables that can easily be assessed in the electronic medical files of primary care facilities in combination with items from history taking and physical examination provided a good to excellent accuracy for detection or exclusion of heart failure in such patients with a C-statistic of 0.82; 95 % CI 0.79–0.86. We rounded the coefficients of the clinical model to the nearest integer after shrinkage to construct this diagnostic algorithm. Variables included were age over 75 (1 point), a history of ischaemic heart disease (1 point), a history of transient ischaemic attack or stroke (1 point), dyspnoea or fatigue (2 points), reported ankle oedema or nocturia (1 point), intermittent claudication (1 point), and signs of fluid overload (1 point). Patients with more than three points have a higher risk of heart failure of more than 20 %. Both electrocardiography and natriuretic peptides had independent added value beyond the clinical model and increased the C-statistic to 0.86; 95 % CI 0.83–0.89 [3].

Does this make screening cost-effective? Annual screening for heart failure of patients with T2DM aged ≥ 60 appears to be cost-effective. A model with information from the electronic medical records (age and comorbidities) and suggestive symptoms of heart failure performed best for a low willingness-to-pay threshold of€ 20,000 per quality-adjusted life-year, a commonly used threshold in Europe. The potential cost-effectiveness would be better if convincing mortality-reducing treatment for HFpEF were to become available in the near future [4].

Already at the time of screening, both screen-detected HFrEF and HFpEF were associated with a clinically relevant lower health status than patients with T2DM without heart failure. This persisted during the 1-year follow-up period. Patients with T2DM without screen-detected or known heart failure had a similar health status to age- and gender-matched subjects from the population at large [4, 5]. Patients with T2DM and screen-detected heart failure also had a worse prognosis than T2DM patients without such a diagnosis. After adjustment for age and gender, the hazard ratio for all-cause mortality was 1.5 (95 % CI 0.8–2.7), for cardiac hospitalisations 2.2 (95 % CI 1.5–3.3), and for the composite endpoint combining these two 1.8 (95 % CI 1.3–2.6). The negative prognostic effect was most evident in those with HFrEF. The hazard ratio adjusted for age and gender for the combined endpoint of all-cause mortality and cardiac hospitalisations was 3.7 (95 % CI 2.2–6.3) for HFrEF and 1.5 (95 % CI 1.0–2.2) for HFpEF compared with those without screen-detected heart failure [6]. Note the mean NT-proBNP at diagnosis was significantly higher for patients with HFrEF than for patients with HFpEF: 104 vs. 33 pmol/l respectively; *p* < 0.001.

## Screening patients with T2DM older than 60 years for heart failure

It seems reasonable to distinguish between screening for HFrEF and HFpEF, because both the prevalence of unrecognised disease and the availability of evidence-based interventions for reducing morbidity and mortality differ considerably. The prevalence is much higher for HFpEF, while convincing evidence-based therapy is only available for HFrEF.

When the screening criteria of Wilson and Jungner are applied, it seems that most criteria for screening are fulfilled for HFrEF, but not for HFpEF (Table 1; [7]). For HFpEF, there is much more uncertainty. For example, its natural history is less clear, and also the underlying pathophysiological pathways have not been fully elucidated, although recently the understanding of possible causal mechanisms has increased [8]. Important questions remain, e.g. about the exact mechanisms involved in the development of ‘diabetic cardiomyopathy’ [9]. Furthermore, there is still an ongoing discussion on the exact echocardiographic criteria that should be fulfilled to establish HFpEF, and this hampers the diagnosis. Finally, treatment of HFpEF is focused on reduction of symptoms of fluid overload and comorbid conditions [10]. Diuretics are the only option for symptom relief, but their prognostic effects have never been adequately evaluated. Other drugs, including beta-blockers, angiotensin-converting-enzyme inhibitors, angiotensin II receptor blockers and mineralo-corticoid inhibitors have been tested in randomised trials in patients with HFpEF, mainly in addition to diuretics, but with disappointing results, with at best a statistically non-significant relative risk reduction on all-cause mortality of around 10 % [10]. These results can be further ‘downgraded’ because a substantial number of the included patients had a left ventricular ejection fraction in the range of 40–50 %, and many consider this to be HFrEF, and not HFpEF. Thus, screening all T2DM patients aged ≥ 60 for HFrEF seems more reasonable according to the criteria of Wilson and Jungner than screening for HFpEF. Although convincing prognostically beneficial therapy for HFpEF is currently not yet available, one could argue that detecting HFpEF is useful. First of all, patients are correctly diagnosed and this prevents misclassification in other diseases that may also cause breathlessness and fatigue, such as COPD. Also, the possibility of adequately treating symptoms of breathlessness by managing incidental periods of volume overload with diuretics should not be undervalued. Finally, one could argue that the patient should at least be aware that he or she has a condition with a relatively poor prognosis.

**Table 1:** Principles of early detection of, or screening for disease of Wilson and Jungner, 1968, applied to detection of heart failure in patients with T2DM aged 60 years or over

	Criteria met for	
	HFrEF	HFpEF
1. The condition sought should be an important health problem	+	+
2. There should be an accepted treatment for the disease	+	+/−
3. Facilities for diagnosis and treatment should be available	+/-	+/−
4. There should be a recognisable latent or early symptomatic stage of the disease	+	+/−
5. There should be a suitable test or examination for the disease	+	+/−
6. The test should be acceptable to the population	+	+/−
7. The natural history of the disease should be adequately understood	+	+/−
8. There should be an agreed policy on whom to treat as patients	+	+/−
9. The total cost of finding a case should be economically balanced in relation to medical expenditure as a whole	+	−
10. Case-finding should be a continuous process, not just a ‘once and for all’ project	+	+/−

*HFrEF* heart failure with reduced ejection fraction, *HFpEF* heart failure with preserved ejection fraction, *+* fully met; *+/−* partly met, *−* does not meet the criterion.

## Time for a change

In many countries, including the Netherlands, T2DM patients are enrolled in disease management programs with a trained nurse practitioner playing a key role. Such programs are usually organised within the primary care setting. Routine assessments recommended in the current T2DM guidelines are: (1) glucose control, (2) blood pressure and lipid control, (3) early detection of retinopathy, (4) prevention of foot ulcers and (5) cardiovascular disease risk control and lifestyle management. See also Table 2.

Apart from the contents of routine assessments in patients with T2DM, also the frequency of such assessments continues to be discussed. The proposed frequency ranges from 1 to 4 times a year; however, a solid evidence-base for either of these recommendations is lacking [11–13]. Especially the high frequency of routine glucose measurements applied in current disease management programs has been questioned [14, 15]. Altogether, there seems to be room for substitution of care in the current disease management programs for T2DM patients. Less frequent routine consultations during the year, with less glucose measurements, and less frequent examinations of the feet and eyes, would create time for paying more attention to uncover latent cardiovascular disease, including heart failure.

**Table 2:** Recommendations for routine consultations of patients with type 2 diabetes in recently updated (inter)national guidelines

Guidelines	Glucose/HbA1c levels/general aspects	Lipids/blood pressure/nephropathy	Foot-control/neuropathy	Eye screening	Cardiovascular disease/risk
American Diabetes Association guidelines, update [11]	Determine HbA1c level at least twice a year	No clear recommendations	Perform an annual comprehensive foot examination to identify risk factors predictive of ulcers and amputations	Eye exam should be performed at least initially and at intervals (not further specified)	Cardiovascular risk factors (dyslipidaemia, hypertension, smoking, a positive family history of premature coronary disease, and the presence of albuminuria) should be assessed at least annually
Dutch GP guidelines on diabetes, update [13]	Three monthly control by a trained nurse practitioner:	Three monthly body weight and blood pressure Annually laboratory testing for serum creatinine, eGFR, potassium, and urine albumin/creatinine ratio or albumin concentration	Three monthly inspection of feet to identify early signs of ulceration Annually neuropathic symptoms, and/or sexual dysfunction	Referral for fundus control 2-yearly and in patients with low degree of diabetic retinopathy annually [20, 21]	Annually assess presence of cardiovascular symptoms (which symptoms not further specified) and discuss lifestyle issues
	Well-being, the occurrence of episodes of hypoglycaemia or hyperglycaemia, problems with keeping to dietary and exercise recommendations, a check on the diabetes medication and adherence to drugs.				
	Measurement of the fasting blood glucose level/HbA1C				
NICE [16]	2–6-monthly intervals until the blood glucose level is stable on unchanging therapy. 6-monthly assessments once the blood glucose level and blood glucose lowering therapy are stable	BP measurement at least annually in a person without previously diagnosed hypertension or renal disease	Make a formal enquiry annually about the development of neuropathic symptoms causing distress	Arrange or perform eye screening at, or around, the time of diagnosis	Review cardiovascular risk status annually by assessment of cardiovascular risk factors, including features of the metabolic syndrome and waist circumference, and change in personal or family cardiovascular history
	Annually: self-monitoring skills	Measure serum creatinine and estimate the glomerular filtration rate once a year	Review the issue of erectile dysfunction in men annually	Arrange repeat of structured eye surveillance annually	If the person is considered not to be at high cardiovascular risk, estimate annually using the UK Prospective Diabetes Study (UKPDS) risk engine [17]
	Offer and reinforce preventive lifestyle advice				
European Association of Diabetes [18]	Glycaemic targets and glucose-lowering therapies must be individualised	No clear recommendations	No clear recommendations	No clear recommendations	No clear recommendations
	Diet, exercise and education remain the foundation of any type 2 diabetes management program				
ESC-EASD [19]	(Intensive) glycaemic control should be appropriately applied in an individualised manner taking into account age, duration of T2DM and history of CVD.	Measure BP annually	No clear recommendations	No clear recommendations	Annual cardiovascular risk assessment and lifestyle management including diet and exercise

## Changing clinical practice

In view of the recent findings in the Netherlands, it seems worthwhile to merge specific screening for heart failure with the existing T2DM disease management programs. The high prevalence of unknown heart failure in patients aged ≥ 60 years, their reduced health status and prognosis justifies such merging. Nurse practitioners could specifically ask about shortness of breath or reduced exercise tolerance, ankle oedema, and nocturia. In suspected cases, the general practitioner could then check for signs of fluid overload (i.e. pulmonary crepitations, but also elevated jugular venous pressure and ankle oedema) followed by a blood test for B-type natriuretic peptide, and/or electrocardiography. Or, alternatively immediate referral for echocardiography [3].

## Conclusions

Early detection of heart failure deserves more attention in the disease management programs of patients with T2DM. Specific screening could be merged with these programs.

To allow for an evidence-based choice of the frequency of screening for heart failure in T2DM patients or subgroups of these patients, cohort studies assessing the incidence of heart failure are required. Also, the exact aetiology of HFpEF should be unravelled because it could enable future prognostically beneficial therapy in these patients. General practitioners, internists and other health care professionals caring for these patients should become aware of the large risk of unknown heart failure in T2DM patients aged ≥ 60 years.

### Funding

Fonds NutsOhra (0702086) and Stichting Stoffels Hornstra.
